# Race/ethnicity and disease severity in IgA nephropathy

**DOI:** 10.1186/1471-2369-5-10

**Published:** 2004-09-02

**Authors:** Yoshio N Hall, Eloisa F Fuentes, Glenn M Chertow, Jean L Olson

**Affiliations:** 1 Division of Nephrology, Department of Medicine, University of California San Francisco, San Francisco, CA, USA; 2 Department of Pathology, University of California San Francisco, San Francisco, CA, USA

## Abstract

**Background:**

Relatively few U.S.-based studies in chronic kidney disease have focused on Asian/Pacific Islanders. Clinical reports suggest that Asian/Pacific Islanders are more likely to be affected by IgA nephropathy (IgAN), and that the severity of disease is increased in these populations.

**Methods:**

To explore whether these observations are borne out in a multi-ethnic, tertiary care renal pathology practice, we examined clinical and pathologic data on 298 patients with primary glomerular lesions (IgAN, focal segmental glomerulosclerosis, membranous nephropathy and minimal change disease) at the University of California San Francisco Medical Center from November 1994 through May 2001. Pathologic assessment of native kidney biopsies with IgAN was conducted using Haas' classification system.

**Results:**

Among individuals with IgAN (N = 149), 89 (60%) were male, 57 (38%) white, 53 (36%) Asian/Pacific Islander, 29 (19%) Hispanic, 4 (3%) African American and 6 (4%) were of other or unknown ethnicity. The mean age was 37 ± 14 years and median serum creatinine 1.7 mg/dL. Sixty-six patients (44%) exhibited nephrotic range proteinuria at the time of kidney biopsy. The distributions of age, gender, mean serum creatinine, and presence or absence of nephrotic proteinuria and/or hypertension at the time of kidney biopsy were not significantly different among white, Hispanic, and Asian/Pacific Islander groups. Of the 124 native kidney biopsies with IgAN, 10 (8%) cases were classified into Haas subclass I, 12 (10%) subclass II, 23 (18%) subclass III, 30 (25%) subclass IV, and 49 (40%) subclass V. The distribution of Haas subclass did not differ significantly by race/ethnicity. In comparison, among the random sample of patients with non-IgAN glomerular lesions (N = 149), 77 (52%) patients were male, 51 (34%) white, 42 (28%) Asian/Pacific Islander, 25 (17%) Hispanic, and 30 (20%) were African American.

**Conclusions:**

With the caveats of referral and biopsy biases, the race/ethnicity distribution of IgAN differs significantly from that of other major glomerulonephridities. However, among individuals undergoing native kidney biopsy, we see no evidence of a race/ethnicity association with severity of disease in IgAN by clinical and IgAN-specific histopathologic criteria. Further studies are needed to identify populations at higher risk for progressive disease in IgAN.

## Background

IgA nephropathy (IgAN) is the most common form of glomerulonephritis (GN) worldwide [1]. Approximately 20–30% of individuals with IgAN develop end-stage renal disease (ESRD) by 10–20 years following diagnosis [2]. Despite the increasing recognition of IgAN as a significant cause of chronic kidney disease (CKD), no specific therapies for IgAN have been developed. Glucocorticoids and fish oil have been recommended for selected patients with IgAN based on controversial results from small clinical trials [3-5]. Many patients with IgAN receive no specific therapy.

Clinical reports suggest that individuals of Asian/Pacific Islander heritage are more likely to be affected by IgAN than whites, African Americans, and persons of Hispanic descent. Reports from U.S. centers have generally compared results of white and African American IgAN patients, with little or no available information on U.S. patients of Asian/Pacific Islander heritage. Studies from Japan and China have reported that more individuals with ESRD in these countries had IgAN, implying that IgAN may have a more severe disease course in certain Asian populations [6,7].

To explore whether IgAN was more common and severe among Asian/Pacific Islanders in our population, we examined clinical and pathologic data on 149 patients with IgAN and a random sample of 149 patients with other primary glomerular lesions (focal segmental glomerulosclerosis, membranous nephropathy and minimal change disease) at the University of California San Francisco (UCSF) Medical Center.

## Methods

The records of 183 percutaneous native and transplant kidney biopsies with a diagnosis of IgAN received between November 1994 and May 2001 at the Renal Pathology Laboratory at UCSF were reviewed. Baseline demographic and clinical data included age, gender, race or ethnicity, history of kidney transplant, date of biopsy, and serum creatinine concentration at the approximate time of biopsy. In addition, the presence or absence of heavy proteinuria (≥ 3.0 g/day, with or without the nephrotic syndrome) and the presence or absence of hypertension at the time of biopsy were recorded. Major ethnic groups included white, Asian/Pacific Islander, Hispanic, African American, and other/unknown. Ethnicity was determined using information from patient health insurance forms and history provided at the time of biopsy. Any case in which Henoch-Schönlein purpura, systemic lupus erythematosus or chronic liver disease were considered likely diagnoses were excluded, as were cases of IgAN superimposed on a systemic disease involving the kidney (e.g., diabetic nephropathy). Two examiners unaware of the clinical data independently reviewed the biopsies. Biopsies displaying fewer than six glomeruli by light microscopy or insufficient immunofluorescence staining, as defined below, were also excluded. Fifteen biopsies were additionally excluded due to incomplete recovery of microscopic slides from files. Biopsies from 149 patients, including 25 kidney transplant recipients, satisfied the criteria for inclusion and provided the basis for the IgAN analytic sample.

Aside from IgAN, the most commonly diagnosed primary glomerular lesions at our institution over the same time period were focal segmental glomerulosclerosis (N = 314), membranous nephropathy (N = 197) and minimal change disease (N = 147). To establish baseline race/ethnicity prevalences for our region and referral population, we collected demographic data on a computer-generated random sample of individuals with non-IgAN glomerular disease (N = 149), stratified by kidney transplant.

### Pathologic assessment

Pathologic assessment of the IgAN native kidney biopsies was performed based on Haas' IgA nephropathy classification system [8]. All cases included in the study also met the following criteria: (1) immunofluorescence studies showing at least 2+ (scale 0 to 3+) mesangial deposition of IgA, with IgA comprising the dominant immunoglobulin deposited in the glomeruli, and (2) electron microscopy (EM) studies showing the presence of mesangial deposits.

### Statistical analysis

Demographic and clinical data are reported as mean ± standard deviation, medians with interquartile ranges, and proportions with 95% confidence limits. Inter-ethnicity comparisons were performed using the Cochrane-Mantel-Haenzsel χ^2 ^test for categorical variables, and analysis of variance (general linear models) or Kruskal-Wallis test for continuous variables. Two-tailed P-values <0.05 were considered statistically significant. SAS version 8.2 was used for all statistical analyses (SAS Institute, Cary, NC, USA).

## Results

Patient clinical characteristics for the IgAN group are summarized in Table 1. Eighty-nine (60%) patients were male and 60 (40%) were female. Fifty-seven (38%) patients were white, 53 (36%) Asian/Pacific Islander, 29 (19%) Hispanic, 4 (3%) African American and 6 (4%) were of other or unknown race/ethnicity. The mean age of the IgAN patients at the time of kidney biopsy was 37 ± 14 years. Among the three main ethnic groups (whites, Hispanics, and Asian/Pacific Islanders), Hispanic patients tended to be slightly younger at the time of biopsy compared with whites and Asian/Pacific Islanders. The distributions of age and gender, however, did not differ significantly among white, Hispanic, and Asian/Pacific Islander groups.

**Table 1:** Summary characteristics: IgA nephropathy

Characteristic^a^	Total (N = 149)	White (N = 57)	Asian/PI^b ^(N = 53)	Hispanic (N = 29)	African American (N = 4)	Unknown (N = 6)	*P*-value^c^
Mean age (yr)	37 ± 14	38 ± 15	37 ± 15	34 ± 14	26 ± 22	36 ± 24	0.54
Mean SCr (mg/dL)	3.1 ± 3.8	3.0 ± 2.6	3.1 ± 4.5	2.9 ± 4.2	6.3 ± 6.2	2.7 ± 2.9	0.96
Male, N (%)	89 (60)	37 (65)	29 (55)	16 (55)	3 (75)	4 (67)	0.50
Proteinuria ≥ 3g/d, N (%)	66 (44)	28 (49)	20 (37)	13 (45)	3 (75)	2 (33)	0.08
Hypertension, N (%)	74 (50)	28 (49)	28 (53)	14 (48)	2 (50)	3 (33)	0.86
Transplant, N (%)	25 (17)	4 (7)	13 (24)	8 (28)	0 (0)	0 (0)	0.15
Haas subclass^d^, N (%)							
I	10 (8)	4 (8)	5 (13)	0 (0)	0 (0)	1 (17)	0.76
II	12 (10)	5 (9)	4 (10)	2 (10)	0 (0)	1 (17)	
III	23 (19)	10 (19)	8 (20)	2 (10)	1 (25)	2 (32)	
IV	30 (24)	13 (24)	8 (20)	7 (33)	1 (25)	1 (17)	
V	49 (40)	21 (40)	15 (37)	10 (47)	2 (50)	1 (17)	

^a^Values represent mean ± standard deviation.

^b^Asian/Pacific Islander

^c^*P*-value refers to overall NOVA, Kruskal-Wallis test, or χ^2 ^for comparison of white, Asian/Pacific Islander and Hispanic groups.

^d^Haas subclass assessment performed on native kidney biopsies (N = 124).

The median serum creatinine (SCr) of the IgAN cohort was 1.7 mg/dL (interquartile range 1.1–3.4 mg/dL). The median SCr of the African American group was signficantly higher (5.0 mg/dL) than the other ethnic groups; however, these calculations were based on a small sample size (N = 4) due to the low prevalence (3%) of African Americans with in our IgAN cohort. Median serum creatinine concentrations were not significantly different among white, Hispanic, and Asian/Pacific Islander groups (*P *= 0.64). Sixty-six patients (44%) exhibited heavy (≥ 3 g/d) proteinuria, and 74 (50%) had documented hypertension (systolic blood pressure ≥ 140 or diastolic blood pressure ≥ 90 mm Hg) at the time of kidney biopsy. The fractions of patients with heavy proteinuria and hypertension at the time of kidney biopsy were not significantly different among white, Hispanic, and Asian/Pacific Islander groups.

Of the 124 native kidney biopsies, the majority of cases (64%) fell into Haas subclasses IV or V, which are known independent predictors of progressive disease and poor renal outcomes [2,8]. Only 22 biopsies (18%) were graded as Haas subclasses I or II, reflecting a relatively high threshold for kidney biopsy in our referral region. The distribution of Haas subclass did not differ significantly among race/ethnicity groups.

Table 2 shows the demographic characteristics of the IgAN and non-IgAN groups. Among the random sample of patients (N = 149) with non-IgAN primary glomerulopathies, 67 (45%) patients had focal segmental glomerulosclerosis (FSGS), 58 (39%) membranous nephropathy, and 24 (16%) minimal change disease. Seventy-seven (52%) patients were male, 51 (34%) white, 42 (28%) Asian/Pacific Islander, 25 (17%) Hispanic, and 30 (20%) were African American. In contrast to previous reports, the distribution of gender did not differ significantly between the IgAN and non-IgAN groups (P = 0.16) [8]. Patients in the non-IgAN group were, however, significantly older (mean age 42 ± 21 years vs. 37 ± 14 years, *P *= 0.006) compared to patients with IgAN. In addition, the distribution of race/ethnicity differed significantly between the two groups (*P *< 0.001). This association of IgAN and distribution of race/ethnicity persisted even when stratified by kidney transplant (*P *< 0.001 for native kidney comparison, *P *= 0.006 for kidney transplant recipient comparison).

**Table 2:** Patient demographics: All glomerular lesions

Characteristic^a^	IgA nephropathy (N = 149)	Non-IgAN glomerular lesions^b ^(N = 149)	*P*-value^c^
Mean age (years)	37 ± 14	42 ± 21	0.006
Male, N (%)	89 (60)	77 (52)	0.16
Transplant, N (%)	25 (17)	25 (17)	1.00
Race/ethnicity, N (%)			
White	57 (38)	51 (34)	<0.001
African American	4 (3)	30 (20)	
Asian/PI^d^	53 (36)	42 (28)	
Hispanic	29 (19)	25 (17)	
Other/Unknown	6 (4)	1 (1)	

^a^Values represent mean ± standard deviation.

^b^Focal segmental glomerulosclerosis, membranous nephropathy, and minimal change disease.

^c^*P*-value refers to overall ANOVA, Kruskal-Wallis test, or χ^2^.

^d^Asian/Pacific Islander

## Discussion

In a biopsy series of 244 patients with IgAN, Haas found fewer African Americans (in a major urban setting), similar to that noted in other U.S.-based studies of IgAN [9-11]. While limited by the size of certain ethnic groups in the study population, Haas found no significant difference in renal survival associated with "white race, black race or Hispanic origin" [8]. The reason for a lower prevalence of IgAN in African Americans relative to other kidney diseases remains unclear. The frequency of IgAN in African Americans does not appear to be influenced by the higher prevalence of the IgA2 allotype among this group [12].

In our study, the fraction of biopsies in subclasses I and II (18%) was similar to that observed by Haas (23%). However, we observed a higher proportion of biopsies in subclasses IV and V (64% vs. 31%), and a lower proportion of biopsies in subclass III (19% vs. 45%) compared with Haas, possibly reflecting a temporal trend towards a higher biopsy threshold along with intergrader measurement bias.

In a study reviewing the pattern of glomerulonephritis in Singapore over the past two decades, Woo and colleagues reported that IgAN was the most common primary GN occurring in Singapore (42% of all primary GNs in the first decade and 45% in the second decade) [13]. In our biopsy population during the same period that we studied, IgAN represented 12.8% of all biopsies with primary glomerular diseases and 8.4% of all biopsies (excluding transplant biopsies for non-glomerular diseases). In China, Li reported that IgAN was the leading cause of ESRD, accounting for approximately 18% of patients [6]. In a national survey of Japanese patients with ESRD, Koyama et al. reported that 28% of new dialysis patients had IgAN listed as their primary cause of ESRD. Moreover, due to the number of additional biopsies showing chronic glomerulonephritis without immunofluorescent microscopic descriptions in the survey, the authors estimated that possibly 40% of newly registered dialysis patients in Japan might have had CKD from IgAN. In contrast, only 0.8% of incident ESRD patients in the U.S. have documented or suspected IgAN [14]. Katznelson and Cecka, using data from the United Network for Organ Sharing (UNOS), have also reported a higher incidence of IgAN and chronic glomerulonephritis causing ESRD in Asian/Pacific Islander American recipients of renal allografts between 1991 and 1996 [15].

In contrast, based on smaller biopsy series, a striking variation in prevalence rates of IgAN has been reported from Europe and South America. In the UK, for example, Ballardie and colleagues noted comparatively low prevalence rates of IgAN in a predominantly white population (Manchester, England) in the early 1970's. In the subsequent 15-year period, however, these investigators reported a phenomenal rise in the observed incidence of IgAN (accounting for 31% of all glomerulopathies in 1986), which the investigators felt more likely reflected a higher frequency of detection rather than true rise in disease incidence. Similar prevalence rates have also been documented from isolated white populations in Finland and southern Italy [16,17]. In contrast, few studies have addressed the epidemiology of IgAN in Latin America. In a small Brazilian single-center cohort (N = 205) of primary glomerular diseases, Mazzarolo et al. reported relatively modest prevalence rates (10.2%) of IgAN [18]. A larger series (N = 1,263) of renal biopsies from Peru noted much lower prevalence rates of IgAN, which accounted for only 0.9% of all glomerular lesions over a 10-year period at a central reference renal pathology laboratory in Lima [19].

These differences may be partially attributed to increased screening and disparities in the indication for kidney biopsy. In Japan and South Korea, for example, school-aged children undergo annual screening for urinary abnormalities; kidney biopsy is subsequently recommended for children with evidence of proteinuria or hematuria [20]. More comprehensive yearly health exams are further performed on full-time salaried employees throughout Japan, Singapore, and Hong Kong, making detection more likely in these ethnic groups. Furthermore, a significant reporting bias may also contribute to the higher reported prevalence rates of IgAN in Asian/Pacific Islanders, e.g., in the study by Koyama et al., only 502 (7%) of the approximate 6800 patients diagnosed with IgAN had undergone a confirmatory kidney biopsy [7].

Although the etiology of IgAN remains unknown, there exists a strong suspicion for an environmental antigen trigger combined with a genetic susceptibility factor. Along these lines, several hypotheses have been proposed to account for the reportedly higher prevalence of IgAN in Asian/Pacific Islanders. With respect to potential dietary antigens, Wakai et al. found that high intake of rice and n-6 polyunsaturated fatty acids (PUFA) were associated with an increased risk of IgAN [21]. Recent reports from Japan have also suggested a potential role of *H. parainfluenzae *as a causative agent of IgAN in Japanese children and adults. Such claims are supported by studies showing the glomerular deposition of outer membrane *H. parainfluenzae *antigens and greater levels of plasma IgA1 antibody against OMHP in Japanese patients with IgAN (compared to Japanese patients with other renal diseases) [22,23]. Whether Japanese, or Asian/Pacific Islanders in general, have higher rates of *H. parainfluenzae *colonization and/or infection has yet to be established.

The presence of either hypertension or proteinuria ≥ 3.0 g/24 hrs at the time of diagnosis significantly correlated with worsened renal survival in IgAN, even when controlling for serum creatinine at the time of kidney biopsy [2]. We found no difference in the distribution of Haas subclass, hypertension and nephrotic proteinuria among Caucasians, Asian/Pacific Islanders, and Hispanics.

Despite ongoing investigative efforts, scant data are available regarding genetic markers that may predispose individuals to progressive disease from IgAN. Recent immunogenetic studies have suggested a potential role for the T-cell receptor (TCR) in the development of immune-mediated diseases. Deenitchina and colleagues found that genetic polymorphism of the TCR constant alpha chain was associated with progression of CKD in a cohort of Japanese patients with IgAN. Although promising, such polymorphisms of the TCR gene have yet to be evaluated in large, prospective studies or by genetic analysis of familial IgAN [24].

Our results contest the assertion that IgAN follows a more severe course in individuals of Asian/Pacific Islander descent. One reason for the similar disease severity of IgAN in our study population may stem in part from the large subpopulation of Filipino patients comprising our Asian/Pacific Islander cohort. It is unclear whether certain subpopulations of Asian/Pacific Islanders, including Filipinos, exhibit IgAN prevalence rates similar to those documented by Koyama and Woo. Anecdotal reports from Thailand and India documenting prevalence rates of 4–9% suggest that IgAN may not have the same epidemiology among all southeast Asians [1]. Despite having higher incidence rates of ESRD than the U.S. white population, the Asian/Pacific Islanders remain a largely unstudied group, for whom more comprehensive data collection is warranted.

There are several important limitations to this report. As with any single-center biopsy series, we may have been underpowered to detect a clinically significant difference due to the limited sample size (type II error). In addition, racial admixture may have also confounded the results, as we were unable to subclassify patients in the Asian/Pacific Islander group or account for the growing population of bi- or multi-ethnic individuals in our population. Furthermore, due to the study's case control design, and breadth of our referral base (northern California and Hawaii), we were unable to control for the criteria for kidney biopsy. As a result, a biopsy bias may have confounded our results. In other words, Asian/Pacific Islander patients in our referral base with mild to moderate proteinuria and/or hematuria might have been given a presumptive diagnosis of IgAN without nephrology referral or confirmatory kidney biopsy. With regard to disease prevalence, these potential referral and biopsy biases based on race/ethnicity are largely conservative in nature, and would have biased our results towards the null. Finally, we have included data from a modest-sized IgAN transplant population (N = 25), the donor demographics of which were unavailable at the time of the study. However, the association of race/ethnicity and distribution of glomerular lesion persisted, even when stratified by kidney transplant, and thus our overall conclusions remained the same. In addition, a small European study of donor-recipient pairs (average follow-up 7 years) has shown that when a donor kidney with asymptomatic IgA deposits is transplanted into a recipient with ESRD secondary to a disease other than IgAN, the IgA immune deposits in the donor kidney are rapidly removed [25].

## Conclusions

In conclusion, with the caveats of referral bias and biopsy bias, the race/ethnicity distribution of IgAN differs significantly from that of other major glomerulonephridities. However, among individuals undergoing native kidney biopsy, we see no evidence of a race/ethnicity association with severity of disease in IgAN by clinical and IgAN-specific histopathologic criteria. Further studies are needed to identify populations at higher risk for progressive disease in IgA nephropathy.

## Competing interests

None declared.

## Authors' contribution

YH designed the study, collected and analyzed the data, and drafted the manuscript. GC supervised the study design, analyzed the data, and edited the manuscript. EF graded the IgAN histopathologic slides. JO collected, reviewed and graded all histopathologic data, and edited the manuscript. All authors approved the final manuscript.

## Pre-publication history

The pre-publication history for this paper can be accessed here:


